# Mr. Alder, in Answer to Dr. Brown

**Published:** 1806-01

**Authors:** Tho. Alder

**Affiliations:** Lincoln


### *To the Editors of the Medical and Physical Journal.*

GENTLEMEN,

AS the very abusive language used by Dr. Brown, in the eighty-first Number of your Journal, must necessarily be applied to me, may I request, as an act of common justice, tice, the insertion of a few observations, in answer to it. Dr. Brown calls all those who differ from him in opinion courters "not of truth but of fame," shakers "of all human testimony," and "guilty of a libel on the common sense and veracity of mankind." Now these are extremely heavy charges, and must be applied to me, as I have published in direct opposition to Dr. Brown: but they are totally without proof, and Dr. Brown has not been so fortunate as to demonstrate, even that *he himself* is right. He says, "the fever which prevailed at Up Park Camp, in Jamaica, during June and July, 1803, was, without doubt, contagious, because the barracks were situated upon an eminence, two miles from the sea, and were not crowded nor filthy; the hospital was exemplary, there were no swamps or sources of putrefaction in the neighbourhood, and it was the healthy season of the year;" but more particularly "because no local cause existed which could produce the disease; the disease went progressively through the battalion from the eastern-most or windward point, (the persons nearest the sick always having been the first who were attacked) and the medical men, the serjeants, the orderly men, and the barbers, were all taken ill with it, and either died or recovered with difficulty." He not only upon these facts ventures to say, "the disease was *without doubt* contagious," but goes so far as to maintain, "all comment" upon this communication "is superfluous," and that all who differ from him are what I have just represented, and worse.

Now, gentlemen, I will grant that June and July are in general the healthiest months in Jamaica; yet, does it follow hence, they always must be perfectly healthy? We know the reverse is the case, and that our West India Islands are very unfavourable to European constitutions. I will suppose the barracks, the hospital, and every thing noticed as favourable to health as they could be in Jamaica; yet we know that the air of Jamaica is so bad, that metals cannot be kept from rust in it; and that (from circumstances which I am about to name) this bad air may affect *any place at any time*. There is a wind in the West India Islands from the céntre of the islands individually, towards the sea, every night; and from the sea easterly every day; and Jamaica is only about one hundred miles to the leeward of St. Domingo: of consequence, marshes, or other sources of putrefaction, situated towards the middle of Jamaica, may affect a camp or hospital situated near the sea during the night; and the bad air from St. Domingo, or even from a very large tract of country in Jamaica,

Jamaica, (if any be generated in it) may affect such during the day; that is, if such camp or hospital be situated near Kingston, (which those under present consideration I think are) any effluvium generated in the island for more than fifty miles to the eastward, would certainly reach it every day.

The other circumstances, those, namely, on which Dr. Brown lays particular emphasis, scarcely deserve our attention: no cause strictly local may have existed: the disease, indeed, going from east to west so regularly, seems to show the air which caused it blew in that direction, rather than that contagion went in that course; for the medical men, barbers, and others, would have carried contagion (had any been present) in many directions. The medical men, the orderlies, and the barbers, being taken ill, and many of them dying, proves nothing, for it seems almost the whole battalion was affected; and those even most likely to be so, who were about the sick, from several circumstances, but perhaps, above many, from that fear which Dr. Brown (I judge from his opinion now made public) could not but have inspired them with.

It appears to me, it would have been commendable in Dr. Brown to have occupied his paper with a description of the service the men taken ill had been employed on? what had been their mode of life? what was their habit of body? and whether they had lately come from a different climate or situation? Though, according to the view I have taken of circumstances, these are of less importance; for about the year 1799, a regiment went into barracks in the West Indies (I think in Jamaica) after much exercise and the liberal use of rum; (I think they had got somewhat inured to the climate) all who were bled immediately on the change from action to rest remained well, the rest were seized with the yellow fever; this fact was given me by an officer (a gentleman of veracity) in the regiment; many circumstances will assist bad air in producing fever, and will enable such bad air to do this, as without them would not have done it.

Will you allow me to take this opportunity to acknowledge two mistakes that have slipt into my "Outlines;" the one at page VII of the Introduction, in a note, and another at page XIII of the Preface. Dr. Desgenettes undoubtedly believed the plague was neither contagious nor infectious, when he inoculated himself with the matter of a bubo; but it appears he afterwards changed his mind. The mistake mistake in the Preface is anatomical, and easily seen; I think it consists chiefly in the word "arteries" being inserted instead of "veins."

I am, &c.

*Lincoln, Dec.* 5, 1805. THO. ALDER.

